# Cardiovascular Effects and Benefits of Exercise

**DOI:** 10.3389/fcvm.2018.00135

**Published:** 2018-09-28

**Authors:** Matthew A. Nystoriak, Aruni Bhatnagar

**Affiliations:** Division of Cardiovascular Medicine, Department of Medicine, Diabetes and Obesity Center, Institute of Molecular Cardiology, University of Louisville, Louisville, KY, United States

**Keywords:** physical activity, endothelium, blood flow, atherosclerosis, coronary artery disease

## Abstract

It is widely accepted that regular physical activity is beneficial for cardiovascular health. Frequent exercise is robustly associated with a decrease in cardiovascular mortality as well as the risk of developing cardiovascular disease. Physically active individuals have lower blood pressure, higher insulin sensitivity, and a more favorable plasma lipoprotein profile. Animal models of exercise show that repeated physical activity suppresses atherogenesis and increases the availability of vasodilatory mediators such as nitric oxide. Exercise has also been found to have beneficial effects on the heart. Acutely, exercise increases cardiac output and blood pressure, but individuals adapted to exercise show lower resting heart rate and cardiac hypertrophy. Both cardiac and vascular changes have been linked to a variety of changes in tissue metabolism and signaling, although our understanding of the contribution of the underlying mechanisms remains incomplete. Even though moderate levels of exercise have been found to be consistently associated with a reduction in cardiovascular disease risk, there is evidence to suggest that continuously high levels of exercise (e.g., marathon running) could have detrimental effects on cardiovascular health. Nevertheless, a specific dose response relationship between the extent and duration of exercise and the reduction in cardiovascular disease risk and mortality remains unclear. Further studies are needed to identify the mechanisms that impart cardiovascular benefits of exercise in order to develop more effective exercise regimens, test the interaction of exercise with diet, and develop pharmacological interventions for those unwilling or unable to exercise.

## Introduction

Cardiovascular disease (CVD) is the leading cause of morbidity and mortality worldwide. In the United States, CVD accounts for ~600,000 deaths (25%) each year (1, 2), and after a continuous decline over the last 5 decades, its incidence is increasing again (3). Among the many risk factors that predispose to CVD development and progression, a sedentary lifestyle, characterized by consistently low levels of physical activity, is now recognized as a leading contributor to poor cardiovascular health. Conversely, regular exercise and physical activity are associated with remarkable widespread health benefits and a significantly lower CVD risk. Several long-term studies have shown that increased physical activity is associated with a reduction in all-cause mortality and may modestly increase life expectancy, an effect which is strongly linked to a decline in the risk of developing cardiovascular and respiratory diseases (4). Consistent with this notion, death rates among men and women have been found to be inversely related to cardiorespiratory fitness levels, even in the presence of other predictors of cardiovascular mortality such as smoking, hypertension, and hyperlipidemia (5). Moreover, better fitness levels in both men and women can partially reverse the elevated rates of all-cause mortality as well as CVD mortality associated with high body mass index (6, 7). Recent work from cardiovascular cohorts shows that sustained physical activity is associated with a more favorable inflammatory marker profile, decreases heart failure risk, and improves survival at 30 years follow-up in individuals with coronary artery disease (8–10).

Despite the robust beneficial effects of physical activity and exercise on cardiovascular health, the processes and mechanisms by which frequent physical activity promotes cardiorespiratory fitness and decreases CVD risk remain unclear. In the past several decades, considerable research effort has aimed to identify the major physiological and biochemical contributors to the cardiovascular benefits of exercise, and as a result, significant advances have been made from observational and interventional studies with human participants. In parallel, valuable mechanistic insights have been garnered from experimental studies in animal models. Thus, in this review, we provide a synopsis of the major known effects of exercise and physical activity on principal factors associated with risk for poor cardiovascular health including blood lipids, hypertension, and arterial stiffness. For the purpose of the review, we follow the definition of exercise as “a subset of physical activity that is planned, structured, and repetitive and has as a final or an intermediate objective the improvement or maintenance of physical fitness (11).” These characteristics distinguish exercise from less structured and planned physical activity, which is often not solely for the purpose of maintaining or improving physical fitness. Most long-term observational studies report levels of physical activity, whereas more controlled and short duration studies examine the effects of exercise. Throughout the text, we distinguish between these two types of activities to the extent possible. We also discuss the means by which a healthy cardiovascular system adapts to exercise conditioning as well as recently proposed mechanisms of adaptation that may work to antagonize cardiovascular disease.

## Plasma lipids and atherogenesis

Given the centrality of plasma lipids as key determinants of CVD risk, many studies have tested whether regular engagement in physical activity may lower CVD risk by affecting the levels of circulating lipoproteins. These studies have found that endurance training is associated with elevated levels of circulating high density lipoprotein (HDL) and, to a lesser extent, a reduction in triglyceride levels (12)—both changes that can reduce the risk of coronary heart disease (13). Nonetheless, results concerning the effects of physical activity on plasma lipids have been variable and confounded by an apparent dependence on the type, intensity, and duration of exercise as well as diet (14). In addition, early studies aimed at determining effects of physical activity on low density lipoprotein (LDL) levels did not test the dose-dependence of exercise. However, a study of subjects with mild to moderate dyslipidemia, randomized into high amount/high intensity (23 kcal/kg/wk, jogging), low amount/high intensity (14 kcal/kg/wk, jogging), and low amount/moderate intensity (14 kcal/kg/wk, walking) exercise training groups over a 6 months period, found a dose-dependent effect of exercise on plasma levels of LDL, triglycerides, and large particle, very low density lipoprotein (VLDL) (15). Increasing levels of exercise over time were also found in this study to increase HDL from baseline (pre-exercise regimen) levels. Although higher levels of HDL are associated lower CVD risk (16, 17), recent work suggests that some pharmacological interventions that elevate plasma HDL levels fail to reduce the risk of major cardiovascular events (18, 19). Nevertheless, HDL particle size is a key determinant of ATP binding cassette transporter A1 (ABCA1)-mediated cholesterol efflux (20), indicating that HDL particle size may be an important correlate of CVD risk. Hence, an increase in the size of LDL and HDL particles and a decrease in VLDL particle size, rather than HDL levels *per se*, upon exercise training (15) may impart CVD risk protection. In agreement with this view a recent study investigating the dose-dependent effects of exercise on cholesterol efflux in 2 randomized trials consisting of six distinct exercise doses reported a significant increase in HDL cholesterol and efflux capacity with exercise, albeit in the high amount/high intensity intervention groups only (21). Thus, even though exercise alters plasma lipid profile and increases HDL concentration and particle size, moderate exercise may produce only limited effects on HDL functionality and the contribution of changes in plasma lipoprotein concentration, structure, and function to overall reduction in CVD risk by exercise remains unclear.

In addition to changes in plasma lipids, exercise could directly impact the homeostasis of the arterial wall to antagonize the progression of atherosclerotic disease and thereby contribute to the well-documented reduction in coronary artery disease in people with active lifestyles, when compared with sedentary individuals (22–25). Even in people with symptomatic coronary artery disease, an increase in regular physical activity can improve VO_2_ max and, at high doses (~2,200 kcal/week), promote regression of atherosclerotic lesions (26). In patients with stable CAD, 4 weeks of rowing or cycling led to enhanced vasodilatory responses to acetylcholine, which was associated with increased total endothelial nitric oxide synthase (eNOS) expression and eNOS, and protein kinase B (Akt) phosphorylation (27). That exercise stimulates NO production is supported by animal studies. For instance, it has been reported that carotid arteries from exercised ApoE^−/−^ mice exhibit elevated eNOS expression and suppressed neointimal formation after injury when compared with those from sedentary ApoE^−/−^ control mice (28). In contrast, aorta from sedentary mice kept in normal housing conditions exhibit increased vascular lipid peroxidation and superoxide levels, which may contribute to endothelial dysfunction and lesion formation, when compared with mice subjected to 6 weeks of voluntary wheel running (29). Regular, but not intermittent, physical activity in high cholesterol diet-fed LDLR-null mice has also been found to rescue aortic valve endothelial integrity, reduce inflammatory cell recruitment, and prevent aortic valve calcification (30), which raises the possibility that exercise may reduce the development and progression of degenerative aortic valve disease. Despite this evidence, it remains unclear to what extent salutary changes in blood lipids and vascular function contribute to the cardiovascular benefits of exercise and further studies are required to quantify both lipid-dependent and lipid-independent effects of physical activity.

## Insulin sensitivity

The association between blood lipids and cardiovascular health is highly influenced by systemic insulin sensitivity, and resistance to insulin signaling is known to promote the development of heart disease, in part by altering the blood lipid profile (31). Resistance of adipocytes to the effects of insulin and resulting reduction in glucose uptake leads to the increased release of free fatty acids and greater production and release of triglycerides, and VLDL by the liver (32). In addition, reduced HDL in the insulin resistant state, resulting in part from increased activity of cholesteryl ester transfer protein (CETP), and transfer of cholesteryl esters from HDL to triglyceride-rich lipoproteins (33), suppresses reverse cholesterol transport from the arterial wall and promotes atherosclerotic plaque formation.

Insulin signaling within the vascular endothelium promotes Akt-dependent phosphorylation and activation of eNOS, which produces the vasodilator - NO. This, however, is antagonized by the activation of the Ras-RAF-MAPK pathway that stimulates cell growth and differentiation and increases the production of the potent vasoconstrictor - endothelin-1 (ET-1) (34, 35). During diabetes, selective inhibition of the PI3K-Akt-eNOS pathway, together with compensatory hyperinsulinemia leads to unmasking and stimulation of the MAPK-mediated production of endothelin-1 (ET-1) (36, 37), and vascular smooth muscle proliferation, which could contribute to atherosclerotic plaque development and peripheral artery disease (38, 39). Enhanced endothelial production and secretion of ET-1, along with heightened sympathetic activity may represent key contributing factors in enhanced vasoconstriction of small diameter arteries and arterioles in the insulin-resistant state, thereby increasing systemic vascular resistance to blood flow and elevating arterial blood pressure. In addition, as a hallmark of diabetes and insulin-resistance, elevated blood glucose levels also accelerate the formation of advanced glycation end products (AGEs), proteins and lipids that have undergone non-enzymatic glycation and oxidation, leading to cross-linking of collagen and elastin fibers and loss of vascular compliance (i.e., arterial stiffening) (40, 41).

A number of studies have shown that individuals with insulin-dependent and non-insulin-dependent diabetes mellitus have improved sensitivity to insulin and improved glycemic control after exercise training (42–44). Indeed, it has been found that even a single low-intensity (50% VO_2_ max, 350 kcal expended) exercise session results in significantly improved insulin sensitivity and fatty acid uptake upon examination on the following day (45). Studies in animal models of exercise suggest that increased physical activity can improve insulin sensitivity in adipose tissue, skeletal muscle, and endothelium (46–49), which are major contributors to systemic insulin resistance in individuals with type 2 diabetes. While our understanding of the precise cellular and molecular mechanisms involved in the enhancement of insulin signaling following exercise has been hampered by inconsistent results across species and exercise protocols, it appears that exercise conditioning is associated with adaptive remodeling in the expression or regulation of one or more components of the insulin receptor/insulin receptor substrate (IRS)/PI3K/Akt signaling cascade (50–52). During exercise, insulin levels are slightly reduced and frequently contracting muscle exhibits greater glucose uptake via enhanced insulin-independent sarcolemmal translocation of GLUT4 glucose transporters (53–55). Moreover, muscle damage associated with eccentric exercise can paradoxically cause insulin resistance via TNF-α-mediated reductions in PI3K activity (56–59). Thus, further research is required to elucidate how certain exercise regimens can promote tissue-specific adaptations in insulin-signaling and how these pathways may be targeted to reverse insulin-resistance and associated cardiovascular complications of diabetes.

## Blood pressure

During exercise, increases in cardiac stroke volume and heart rate raise cardiac output, which coupled with a transient increase in systemic vascular resistance, elevate mean arterial blood pressure (60). However, long-term exercise can promote a net reduction in blood pressure at rest. A meta-analysis of randomized controlled interventional studies found that regular moderate to intense exercise performed 3–5 times per week lowers blood pressure by an average of 3.4/2.4 mmHg (61). While this change may appear small, recent work shows that even a 1 mmHg reduction in systolic BP is associated with 20.3 fewer (blacks) or 13.3 fewer (whites) heart failure events per 100,000 person-years (62). Thus, reductions in blood pressure observed when exercise is included as a behavioral intervention along with dietary modification and weight loss (63, 64) could have a significant impact on CVD incidence.

Lower ambulatory blood pressure, associated with chronic aerobic and resistance exercise, is thought to be driven largely by a chronic reduction in systemic vascular resistance (65). Contributing to this effect, shear forces, as well as released metabolites from active skeletal muscle during exercise, signal the production and release of nitric oxide (NO) and prostacyclin from the vascular endothelium, which promotes enhanced vasodilation via relaxation of vascular smooth muscle cells (66). This effect is especially significant because a reduction in eNOS activity that occurs with aging or due to NOS3 polymorphism, has been reported to contribute to hypertension (67–69). Long-term exercise training increases eNOS expression as well as NO production in hypertensive individuals, consistent with the blood pressure lowering effect of physical activity (70). An important role of NO in mediating the vascular effects of exercise is further supported by results showing that rats with hypertension induced by chronic NOS inhibition undergoing a swimming exercise regimen for 6 weeks have significantly elevated eNOS protein expression and improved acetylcholine-induced vasodilation (71). Thus, improvements in NO production and bioavailability appear to represent significant factors that contribute to improved endothelium-dependent vasodilation following exercise training, which can reduce resting vascular resistance and lower blood pressure. However, in addition to NO-mediated reductions in resistance vascular tone, adaptive reductions in sympathetic nerve activity, prevention or reversal of arterial stiffening, and suppression of inflammation are also likely contributors to the blood pressure lowering effects of exercise, although the impact of exercise on these outcomes may be population specific (e.g., at-risk versus healthy adults) (72–74). As with changes in blood lipid profile, it remains unclear to what extent the blood pressure lowering effects of exercise can account for the beneficial effects of exercise on CVD risk and mortality.

## Cardiac adaptations

During exercise, the heart is subjected to intermittent hemodynamic stresses of pressure overload, volume overload, or both. To normalize such stress and to meet the systemic demand for an increased blood supply, the heart undergoes morphological adaptation to recurrent exercise by increasing its mass, primarily through an increase in ventricular chamber wall thickness. This augmentation of heart size is primarily the result of an increase in the size of individual terminally differentiated cardiac myocytes (75). Adaptive remodeling of the heart in response to exercise typically occurs with preservation or enhancement of contractile function. This contrasts with pathologic remodeling due to chronic sustained pressure overload (e.g., during hypertension or aortic stenosis), which can proceed to a loss of contractile function and heart failure (76).

Recent work in experimental animal exercise models has identified several cellular and molecular alterations involved in the physiologic growth program of the heart that accompanies exercise conditioning. Whereas pathologic remodeling of the heart is associated with a reduction in oxidative energy production via fatty acid oxidation and more reliance on glucose utilization, mitochondrial biogenesis and capacity for fatty acid oxidation are enhanced following exercise (77, 78). A recent study suggests that changes in myocardial glycolytic activity during acute exercise and the subsequent recovery period can also play an important role in regulating the expression of metabolic genes and cardiac remodeling (79). Possibly upstream of these metabolic changes, studies have also revealed a dominant role for IGF-1 and insulin receptor signaling, via the PI3K/Akt1 pathway leading to the activation of transcriptional pathways associated with protein synthesis and hypertrophy (80, 81). Untargeted approaches have identified other major determinants of transcriptional programs that drive the exercise-induced hypertrophic response. For instance, it has been reported that exercise-induced reduction in the expression of CCAAT-enhancer binding protein β (C/EBPβ) relieves its negative regulation by CBP/p300-interactive transactivator with ED-rich carboxy-terminal domain-4 (Cited4) (82). Activation of Cited4 has been found to be necessary for exercise-induced cardiac hypertrophy, and cardiac-specific overexpression of the gene is sufficient to increase heart mass and protect against ischemia/reperfusion injury (83). Other transcriptional pathways known to be activated by pathologic stimuli and cardiac hypertrophy, such as NFATc2, are decreased in exercise models (79, 84), suggesting that some signaling pathways activated during exercise-induced growth program may directly antagonize specific factors that promote pathological remodeling.

In addition to metabolic and molecular remodeling, exercise can also promote functional adaptation of the heart, which may ultimately increase cardiac output and reduce the risk of arrhythmia. Clinical studies have shown that exercise-trained individuals have improved systolic and diastolic function (85, 86), while results of studies using animal models of exercise show that endurance exercise promotes enhanced cardiomyocyte contraction-relaxation velocities and force generation (87–90). This effect of exercise on cardiomyocyte contractile function may be related to alterations in the rise and decay rates of intracellular Ca^2+^ transients, possibly due to enhanced coupling efficiency between L-type Ca^2+^ channel-mediated Ca^2+^ entry and activation of subsarcolemmal ryanodine receptors (RyR; i.e., calcium-induced calcium release), and increased expression and activity of the sarcoendoplasmic reticulum Ca^2+^ ATPase (SERCA2a) and sodium-calcium exchanger (NCX) (88, 91, 92). In addition, the sensitivity of the cardiomyocyte contractile apparatus may also become more sensitive to Ca^2+^, thus producing a greater force of contraction at a given [Ca^2+]^_i_, following exercise, (93). These changes may at least partially depend on upregulation of the Na^+^/H^+^ antiporter and altered regulation of intracellular pH.

During pathologic remodeling of the heart, electrical instability can result from a lack of upregulation of key cardiac ion channel subunits associated with action potential repolarization relative to an increase in myocyte size (94). In contrast, increased myocyte size in physiological hypertrophy is associated with the upregulation of depolarizing and repolarizing currents, which may be protective against abnormal electrical signaling in the adapted heart (95, 96). For example, cardiac myocytes isolated from mice after 4 weeks of swim training were found to have elevated outward K^+^ current densities (i.e., I_to,f_, I_K,slow_, I_ss_, and I_K1_) and increased expression of underlying molecular component Kv and Kir subunits in parallel with increases in total protein levels (96). Interestingly, a follow up study found that while increases in K^+^ channel subunit expression following exercise training requires PI3K, these changes occur independently of Akt1 and hypertrophy (97).

## Blood and vasculature

The oxygen carrying capacity of blood, determined by the number of circulating erythrocytes and their associated intracellular hemoglobin concentration, is an important determinant of exercise performance and resistance to fatigue (98). High endurance athletes commonly have “athlete's anemia,” possibly due to loss of erythrocytes, or low hematocrit secondary to an expansion of plasma volume (99). Yet, overall total erythrocyte mass is increased in athletes, especially those who train at high altitude (100). This is in part due to a dose-dependent effect of O_2_ on hypoxia-inducible factor (HIF)-mediated erythropoietin production as well as upregulation of erythropoietin receptors, iron transporters, and transferrins (101). Multiple studies have shown that hematopoiesis is enhanced immediately following exercise (102, 103). Intense exercise is associated with the release of a variety of stress and inflammatory factors that are active on the bone marrow such as cortisol, IL-6, TNF-α, PMN elastase, and granulocyte colony stimulating factor (104–106). Although HPCs appear to modestly decline in the period immediately following an exercise session in conditioned runners, one study found that circulating CD34^+^ hematopoietic progenitor cell counts were 3- to 4-fold higher in runners vs. non-runners at baseline (102), which may represent an adaptive response that facilitates tissue repair. A subsequent study found that a bout of intense exercise was associated with a release of CD34^+^/KDR^+^ endothelial progenitor cells from the bone marrow and that this effect was enhanced in individuals with elevated LDL/HDL and LDL/TC profiles (107). Likewise, a significant increase in the number of circulating EPCs, associated with increased levels of VEGF, HIF-1α, and EPO was found within hours after varying intensities of resistance training in women (108). Nonetheless, the physiological significance of these responses remains unclear, as the effects of exercise on angiogenesis and the wound healing response have not been systematically studied.

The resistance arterial vascular network also undergoes functional and structural adaptation to exercise (109). During acute exercise, small arteries and pre-capillary arterioles that supply blood to the skeletal muscles must dilate to increase blood flow through the release of vasodilatory signals (e.g., adenosine, lactate, K^+^, H^+^, CO_2_) from active surrounding muscle (110–112). Repeated exercise leads to an adaptive response in skeletal muscle arterioles that includes increased vascular density coupled with greater vasodilatory capacity, such that enhanced perfusion can occur after conditioning (113–116). This may be partly due to adaptation of the endothelium to the complex interplay of recurrent variations in hemodynamic stresses and vasodilatory stimuli of exercise. Endothelial synthesis of NO is greatly increased at rest and during exercise in conditioned individuals/animals (117). A similar adaptive response to exercise has also been noted in the coronary vasculature, which must dilate to meet the increased metabolic demands of the myocardium (118). Exercise-trained humans and animals demonstrate reduced myocardial blood flow at rest, which may reflect a reduction in cardiac oxygen consumption primarily as a result of lower resting heart rate (119, 120). However, a large body of evidence suggests that multiple mechanisms converge to enhance the ability of the coronary circulation to deliver a greater supply of oxygen to the conditioned myocardium during exercise. This includes structural adaptations consisting of an expansion in the density of intramyocardial arterioles and capillaries as well as enhanced microvascular collateral formation (121–124). Additionally, like skeletal muscle arterioles, coronary arterial network enhances its responsiveness to vasoactive stimuli via a number of distinct mechanisms including, but not limited to, augmentation of endothelial NO production, altered responsiveness to adrenergic stimuli, or changes in the metabolic regulation of vascular tone (125–127). In addition, some studies implicate hydrogen peroxide (H_2_O_2_)-mediated vasodilation in opposing exertion-induced arterial dysfunction in overweight obese adults after a period of exercise training (128, 129), suggesting enhanced contribution of NO-independent mechanisms to improved microvascular endothelial function with exercise. Collectively, these adaptations may act to support enhanced myocardial function and increased cardiac output during repeated exercise, and increased total body oxygen demand following exercise conditioning. Further advancement of our understanding of how blood flow is improved in response to exercise could lead to novel therapeutic strategies to prevent or reverse organ failure in patients resulting from inadequate blood flow.

## Concluding remarks and remaining questions to be addressed

Despite the extensive body of knowledge documenting the unequivocal health benefits of exercise, a vast majority of Americans do not engage in sufficient physical activity (130). Nonetheless, mortality risk reduction appears with even small bouts of daily exercise and peak at 50–60 min of vigorous exercise each day (131). However, the question remains as to how much exercise is optimal for cardiovascular health benefit. Studies in endurance runners show that the frequency of adverse cardiovascular events in marathoners is equivalent to that in a population with established CHD, suggesting that too much exercise may be detrimental (132). An upper limit for the cardiovascular benefits of exercise is further supported by a recent study showing that individuals who completed at least 25 marathons over a period of 25 years have higher than expected levels of coronary artery calcification (CAC) and calcified coronary plaque volume when compared with sedentary individuals (133). A recent investigation also showed that individuals who maintain very high levels of physical activity (~3 times recommended levels) have higher odds of developing CAC, particularly in white males (134). In contrast, other studies report greater plaque stability due to calcification in exercisers, thus indicating that with higher levels of physical activity, plaque quality may be favorably impacted to lower the risk of cardiovascular events, despite a higher incidence of plaques and normal CAC scores (135, 136). Nevertheless, as with other effects of exercise, the shape of the dose-response curve remains obscure and it is not clear at what levels of intensity and duration the effects of exercise begin to taper and where they start to become detrimental. It is also unknown how this threshold of transition from benefit to harm is affected by personal demographic features such as age, sex, ethnicity, and baseline CVD risk.

**Figure 1:**
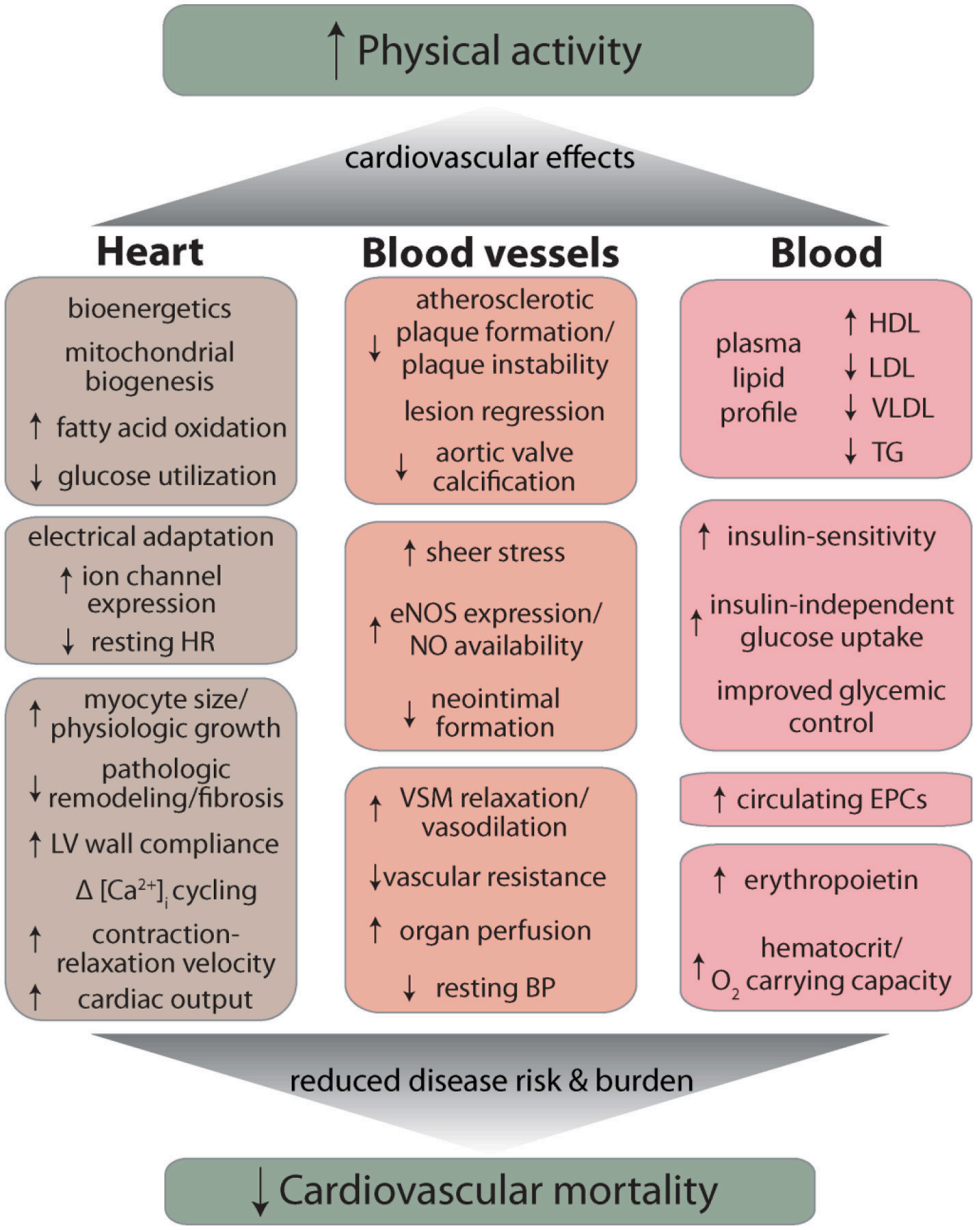
Overview of major cardiovascular effects of exercise. Abbreviations: HR, heart rate; LV, left ventricle; eNOS, endothelial nitric oxide synthase; NO, nitric oxide; VSM, vascular smooth muscle; BP, blood pressure; HDL, high density lipoprotein; LDL, low density lipoprotein; VLDL, very low density lipoprotein; TG, triglycerides; EPC, endothelial progenitor cell.

Other remaining questions are: can initiation of regular exercise, later in life, reverse the consequences of lifestyle choices made during earlier years of life (e.g., sedentarism, smoking), and whether the beneficial effects of exercise show circadian or seasonal dependence such that exercising during a particular time of day or a particular season imparts more benefit than under other conditions. A recent study showing that adherence to a two-year, high-intensity exercise program decreases left ventricular stiffness in previously sedentary middle-aged participants (137) suggests that to some extent, beginning exercise, even late in life can be effective in reversing structural and functional changes in the cardiovascular system associated with aging and/or disease states such as heart failure with preserved ejection fraction. Yet, perhaps the most important questions relate to the mechanisms by which exercise imparts it remarkable benefits to cardiovascular health. As discussed above and summarized in Figure 1, regular physical activity can ameliorate a variety of CVD risk factors such as dyslipidemia or hypertension, but a well-powered analysis of the cardiovascular effects of exercise revealed that reduction in the burden of classical risk factors can account for only about 59% of the total reduction in cardiovascular mortality (138). What accounts for the remaining 41% reduction in risk remains unclear, but it may be related to changes in systemic inflammation as well as favorable responses to acute inflammatory challenge. Indeed exercise has pervasive effects on immune cells—natural killer cells, neutrophils, monocytes, regulatory T cells, as well as the balance of T-cell types are all affected by exercise (139) and it promotes a healthy anti-inflammatory milieu (140). Nevertheless, how exercise affects inflammation and immunity and how these changes could account for the salubrious effects of exercise on cardiovascular disease risk and mortality are important questions that require additional careful investigations. Additional work is also required to assess how nutrition affects exercise capacity as well as the cardiovascular benefits of exercise and how exercise affects the gut and the microbiome (139, 140). In this regard, it is important to clearly delineate the extent to which nutritional supplements such as β-alanine and carnosine, which enhance the buffering capacity of muscle (141) affect exercise capacity as well as muscle growth and hypertrophy. Such work is essential and important not only for a basic understanding of the mechanisms of exercise-induced protection, but also for developing more effective exercise regimens, testing the efficacy of combined treatments involving exercise and dietary supplements, and for devising appropriate pharmacological interventions for those who would not or cannot exercise.

## Author contributions

All authors listed have made a substantial, direct and intellectual contribution to the work, and approved it for publication.

### Conflict of interest statement

The authors declare that the research was conducted in the absence of any commercial or financial relationships that could be construed as a potential conflict of interest.
